# Gene expression in breast and adipose tissue after 12 months of weight loss and vitamin D supplementation in postmenopausal women

**DOI:** 10.1038/s41523-017-0019-5

**Published:** 2017-04-21

**Authors:** Caitlin Mason, Lei Wang, Catherine Duggan, Ikuyo Imayama, Sushma S. Thomas, Ching-Yun Wang, Larissa A. Korde, Anne McTiernan

**Affiliations:** 1 Public Health Sciences Division, Fred Hutchinson Cancer Research Center, Seattle, WA USA; 2 Department of Biostatistics, University of Washington, Seattle, WA USA; 3 Department of Medicine, University of Washington, Seattle, WA USA; 4 Department of Epidemiology, University of Washington, Seattle, WA USA

## Abstract

Adipose tissue is involved in the etiology of postmenopausal breast cancer, possibly through increased sex steroid hormone production, inflammation, and altered adipokines. Vitamin D may affect these pathways but its effect on gene expression in different tissues has not been examined. Within a double-blind, 12-month placebo-controlled randomized trial, we compared 2000 IU/day oral vitamin D_3_ supplementation (*N* = 39) vs. placebo (*N* = 40) on the expression of 5 genes in breast and adipose tissue in overweight/obese postmenopausal women (50–75 years). All participants had serum 25-hydroxyvitamin D (25(OH)D) levels ≥ 10–<32 ng/mL (“insufficient”) and concurrently completed a behavioral weight loss program. Random periareolar fine needle aspiration (RPFNA) and abdominal subcutaneous adipose tissue biopsies were performed at baseline and 12 months. Changes in expression of aromatase (*CYP19A1*), peroxisome proliferator-activated receptor gamma (*PPARG*), adiponectin (*ADIPOQ*), monocyte-chemoattractant protein 1 (*MCP-1*), and vitamin D receptor (*VDR*) were analyzed by qRT-PCR. Compared to placebo, 2000 IU vitamin D did not show significant effects on gene expression in breast or adipose tissue. Replete women (i.e., 25(OH)D ≥ 32 ng/mL; *N* = 17) showed a small decrease in *MCP-1* expression compared to an increase among women who remained ‘insufficient’ despite supplementation (*N* = 12) (Replete:−1.6% vs. Non-replete: 61.2%, *p* = 0.015) in breast, but not adipose tissue. No statistically significant differences in gene expression were detected according to degree of weight loss. Vitamin D repletion during weight loss may have different effects on gene expression in breast and adipose tissue. Further research on the localized effects of vitamin D is needed to determine its effect on breast cancer risk.

## Vitamin D: Supplementation offers glimmer of benefit

Vitamin D supplementation may help lower the expression of a cancer-associated gene in breast tissue, according to a preliminary trial. Anne McTiernan, Larissa Korde and colleagues at the Fred Hutchinson Cancer Research Center in Seattle, Washington, USA, ran a 12-month randomized clinical study in which 79 overweight postmenopausal women received either daily vitamin D pills or a placebo while concurrently completing a behavioral weight loss program. At the end of the year-long trial, vitamin D seemed to have no effect on the expression of five cancer-linked genes in either breast or fat tissues compared to placebo. However, women taking the vitamin supplements whose vitamin D levels went up showed a significant drop in their breast tissue in the expression of one cancer-related gene compared to women whose vitamin levels didn’t rise despite the daily pills.

## Introduction

Obesity is an established risk factor for postmenopausal breast cancer.^1^ Although the mechanisms linking excess body weight and breast cancer risk are not fully understood, proposed mechanisms underlying this association include alterations in the production of sex steroid hormones, insulin resistance, adipose tissue dysfunction and altered cytokine levels that result in a state of chronic low grade inflammation.^2, 3^

Vitamin D insufficiency, which is commonly observed with obesity,^4, 5^ has been inversely associated,^6–8^ although not consistently,^9^ with postmenopausal breast cancer risk in epidemiologic studies and clinical trials. Circulating vitamin D has been shown to have effects on several of the mechanistic pathways linking obesity and breast cancer risk, including leptin,^10^ adiponectin,^11^ insulin resistance,^12, 13^ and markers of inflammation.^14, 15^

The effects of vitamin D are mediated by binding of its active metabolite 1α,25-dihydroxyvitamin D (calcitriol) to the vitamin D receptor (VDR), a member of the nuclear receptor superfamily, which is expressed in adipose tissue and mammary epithelial tissue and shows altered expression in premalignant and malignant lesions of the breast.^16, 17^ The 1α,25-(OH)_2_ D_3_/VDR complex can up-regulate or down-regulate gene expression depending on the target tissue^18^ and has effects on cell division, apoptosis, and contact inhibition.^19, 20^ For example, calcitriol decreases aromatase expression and attenuates estrogen signaling in breast cancer cells,^21^ and breast tumors with greater *VDR* expression relapse more slowly after first diagnosis.^22^ In adipose tissue, VDR regulates metabolism^23^ and calcitriol inhibits adipogenesis by acting on multiple targets including *PPARG* expression and modulating inflammation via reduced expression of inflammatory genes *MCP-1*, *IL-6*, and *IL-8*.^24^ However, human studies of vitamin D and breast cancer risk are frequently limited to measuring peripheral blood biomarkers and few, if any, published data exist on the differential effects of vitamin D on gene expression in target tissues.

**Fig. 1:**
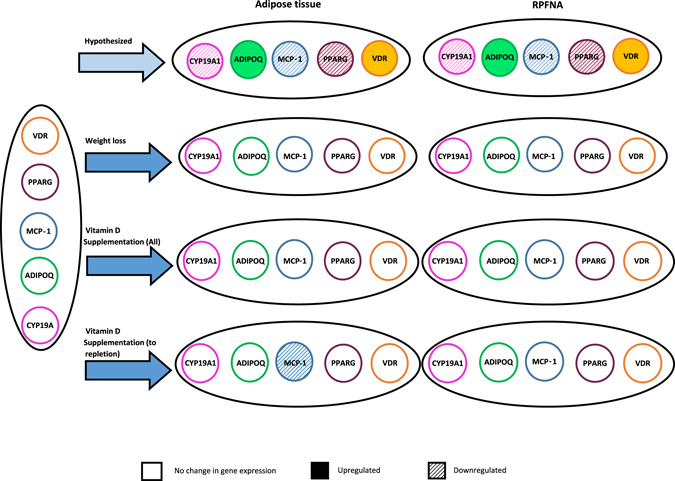
Hypothesized and observed changes in gene expression according to treatment condition in vitamin D, diet and activity (ViDA) study

In the context of a 12-month, double-blinded, placebo-controlled randomized trial, we investigated the effects of oral vitamin D_3_ supplementation (2000 IU/day) vs. placebo on the expression of 5 pre-selected genes in samples of breast random fine needle periaerolar fine needle aspirate (RPFNA) tissue and subcutaneous adipose tissue from a subset of postmenopausal women participating in a diet and exercise weight loss program. These genes were pre-specified for analysis based on their hypothesized involvement in candidate pathways linking adiposity, vitamin D status and breast cancer risk. The genes assayed and the hypothesized changes in expression are shown in Fig. 1. The genes were: *VDR* which mediates the cellular effects of vitamin D and may modulate breast cancer risk;^25^ Aromatase (*CYP19A1*) which regulates the conversion of testosterone to estradiol, and thus would be expected to increase breast cancer risk;^26^ Peroxisome proliferator-activated receptor gamma (*PPARG*), a key mediator of adipogenesis that helps regulate fatty acid storage and glucose metabolism and which is overexpressed in breast tumor cells;^27, 28^ Monocyte chemoattractant protein-1 (*MCP-1*) which regulates migration and infiltration of monocytes/macrophages, and may promote breast tumor development;^29^ and Adiponectin (*ADIPOQ*) which is involved in regulating glucose levels and fatty acid breakdown, and is inversely associated with cancer risk.^30^

## Results

The baseline characteristics of study participants are shown in Table 1. In total, 79 eligible women consented to undergo RPFNA and abdominal subcutaneous adipose tissue biopsy at baseline; however, samples collected from one participant had insufficient yield for analysis resulting in a final sample of 78 women (vitamin D: *N* = 38; placebo: *N* = 40). The majority (92%) were non-Hispanic white; their mean age and BMI were 59.3 ± 5.2 years and 32.8 ± 6.0 kg/m^2^, respectively. At 12 months, 62 (79%) completed follow-up RPFNA and 61 (78%) completed abdominal fat tissue biopsies.

**Table 1:** Baseline characteristics of ViDA participants who consented to abdominal adipose biopsy (AB) and/or random fine needle periareolar aspiration (RPFNA)

	All ViDA trial participants (*N* = 218)			All biopsy participants* (*N* = 78)			Placebo (*N* = 40)			Vitamin D (*N* = 38)		
	*N*	%		*N*	%		*N*	%		*N*	%	
*Ethnicity*												
Non-Hispanic White	190	87.2%		72	92.3%		36	90.0%		36	94.7%	
Non-Hispanic Black	14	6.4%		3	3.9%		2	5.0%		1	2.6%	
Other (American Indian, Asian)	14	6.4%		3	3.9%		2	5.0%		1	2.6%	
*Education*												
College graduate	85	39.0%		21	26.9%		14	35.0%		7	18.4%	
Graduate degree	76	34.9%		29	37.2%		15	37.5%		14	36.8%	
High school or vocational training	7	3.2%		7	9.0%		4	10.0%		3	7.9%	
Some college or associate degree	50	22.9%		21	26.9%		7	17.5%		14	36.8%	
	*N*	MEAN	SD	*N*	MEAN	SD	*N*	MEAN	SD	*N*	MEAN	SD
Age (year)	218	59.6	5.1	78	59.3	5.2	38	59.3	5.6	40	59.3	4.8
Weight (kg)	218	87.7	16.3	78	89.6	16.8	38	89.1	16.1	40	90.2	17.7
BMI (kg/m^2^)	218	32.4	5.8	78	32.8	6.0	38	32.8	5.5	40	32.7	6.4
Waist circumference (cm)	218	100.1	12.3	78	101.7	13.5	38	101.6	12.3	40	101.8	14.6
Hip circumference (cm)	218	116.6	12.2	78	118.0	13.2	38	118.4	12.3	40	117.6	14.1
Body fat (kg)	215	41.6	10.4	76	42.5	10.4	37	42.1	10.3	39	42.8	10.7
Body fat (%)	215	47.4	4.9	76	47.7	5.1	37	47.7	5.2	39	47.7	5.1
Lean mass (kg)	215	41.4	5.8	76	41.7	5.9	37	41.3	5.4	39	42.1	6.4
Pedometer steps/day (7 d average)	214	5629	3263	76	5324	2933	36	5741	3204	40	4949	2651
Moderate to vigorous activity (min/week)	218	142	143	78	148	143	38	112	105	40	181	165
Average caloric intake (kcal/day)^a^	206	2004	699	73	1946	677	37	1971	760	36	1920	590
Relative % calories from fat	206	33.0	6.2	73	34.3	6.8	37	35.4	7.8	36	33.1	5.5
Relative % calories from protein	206	17.6	3.2	73	17.8	3.6	37	17.1	3.0	36	18.5	4.0
Relative % calories from carbohydrate	206	48.3	7.4	73	47.5	7.7	37	48.3	8.0	36	46.5	7.5
Estrone (pg/ml)	218	44.6	19.8	78	42.8	18.2	38	40.6	18.5	40	45.0	17.8
Estradiol (pg/ml)	218	15.5	19.4	78	12.7	6.2	38	12.4	6.5	40	12.9	5.9
Leptin (ng/mL)	218	40.3	18.9	78	41.2	19.8	38	41.4	19.4	40	40.9	20.3
Adiponectin (μg/mL)	218	12.4	6.1	78	12.0	5.4	38	12.4	5.3	40	11.6	5.6
IL_6 (pg/ml)	217	7.2	19.8	78	5.7	5.8	38	5.7	5.6	40	5.7	6.1
TNFa (pg/ml)	217	10.4	4.7	78	9.9	4.0	38	9.7	4.1	40	10.2	3.9
IL_10 (pg/ml)	217	53.7	386.6	78	22.9	22.8	38	20.2	17.2	40	25.4	27.0
IL_8 (pg/ml)	217	13.5	64.9	78	13.9	62.0	38	20.9	88.8	40	7.2	3.8
Insulin (µu/ml)	218	12.5	6.8	78	12.6	6.3	38	12.6	6.2	40	12.7	6.5
C-reactive protein (mg/ml)	218	4.7	8.7	78	3.8	3.9	38	3.9	3.9	40	3.7	4.1
Serum 25(OH)D (ng/mL)	218	21.4	6.1	78	20.5	6.5	38	21.6	6.1	40	19.4	6.8

* 1 participant consented to undergo biopsies but had insufficient sample to be included in analyses and is not included here

^a^ Values derived from FFQ were truncated <600 kcal and >4000 kcal

Cytology was assessed on the first 37 RPFNA samples. Baseline characteristics of these 37 participants were similar to those noted above. In this population of postmenopausal women, only 27% of samples contained epithelial cells; two samples contained sufficient cells for cytologic analysis (100–500 cells). For samples with epithelial cells, age was associated with higher cell count (mean age = 55.9 for participants with >50 epithelial cells vs. 60.2 for participants with <50 epithelial cells, *p* = 0.05). Because of low yield, cytology was not assessed on additional samples.

Serum 25(OH)D increased a mean of 13.6 ng/mL in the vitamin D arm and decreased a mean of 1.1 ng/mL in the placebo arm over 12 months (*p* < 0.001). The mean weight change was −6.8% in both the vitamin and placebo arms (*p* > 0.05). Statistically significant correlations between baseline serum biomarkers and gene expression were consistently observed suggesting that our measures of gene expression provide good agreement with circulating levels of blood biomarkers (Supplementary Table 1).

Compared to placebo, women receiving 2000 IU vitamin D did not show any statistically significant changes in expression of the selected genes in either breast RPFNA tissue or abdominal subcutaneous adipose tissue (Table 2). Results were similar when this analysis included all available data compared to an analysis limited only to women with complete baseline and 12-month data. Similarly, no statistically significant differences in gene expression were detected across categories of weight loss (results not shown).

**Table 2:** Twelve-month change in gene expression (unsupervised) in women randomized to daily vitamin D (2000 IU) vs. placebo

	Adipose tissue			RPFNA		
Gene name	Vit. D	Placebo	*p*	Vit. D	Placebo	*p*
	(*n* = 38/28)^a^	(*n* = 40/33)		(*n* = 38/29)	(*n* = 40/33)	
*VDR*						
Baseline	0.29 (0.23)	0.23 (0.20)		0.82 (0.67)	0.59 (0.52)	
12-Month follow-up	0.27 (0.24)	0.20 (0.18)		0.72 (0.57)	0.56 (0.76)	
Difference	−7.3%	−10.5%	0.503	−12.9%	−5.1%	0.259
*CYP19A1*						
Baseline	3.43 (2.30)	3.10 (2.72)		0.91 (0.69)	1.17 (1.60)	
12-Month follow-up	2.77 (2.22)	2.61 (1.76)		1.25 (1.66)	1.06 (1.00)	
Difference	−19.1%	−15.8%	0.864	36.9%	−9.0%	0.301
*PPARg*						
Baseline	16.39 (4.75)	15.52 (4.27)		8.38 (4.79)	8.01 (5.20)	
12-Month follow-up	17.97 (4.17)	16.63 (4.40)		11.09 (5.61)	9.91 (5.56)	
Difference	9.7%	7.1%	0.692	32.4%	23.8%	0.585
*ADIPOQ*						
Baseline	31.58 (7.19)	32.20 (9.66)		14.92 (8.19)	14.65 (9.45)	
12-Month follow-up	32.45 (7.87)	32.73 (8.30)		16.92 (10.25)	15.48 (8.42)	
Difference	2.7%	1.6%	0.689	13.4%	5.7%	0.627
*MCP-1*						
Baseline	68.72 (33.13)	67.43 (34.32)		32.37 (43.74)	44.82 (65.07)	
12-Month follow-up	83.41 (51.68)	90.74 (53.44)		41.52 (37.63)	37.11 (27.71)	
Difference	21.4%	34.6%	0.802	28.3%	−17.2%	0.605

GEE model comparing the 12-month change between vitamin D vs. placebo; all available data, unadjusted

Difference: [(mean_post-mean_pre)/mean_pre]*100%

*RPFNA* random periareolar fine needle aspiration

^a^ (*n* = baseline sample size/12 month follow-up sample size)

Correlations between 12-month gene expression changes in abdominal adipose tissue and breast RPFNA tissue are shown in Table 3. Only the correlation between the 12-month change in *CYP19A1* expression in breast and abdominal adipose tissue in the vitamin D arm reached statistical significance (*r* = 0.44, *p* = 0.018). All other correlations were weak (*r* = −0.010 to 0.307, all *p* > 0.10).

**Table 3:** Associations (Pearson correlation coefficients) between 12-month changes in gene expression in RPFNA and abdominal adipose tissue

Vitamin D (2000 IU/day) Arm (*N* = 28)						
		12-month gene expression changes in breast tissue (RPFNA)				
		*ADIPOQ*	*CYP19A1*	*VDR*	*PPARg*	*MCP-1*
12-Month gene expression changes in abdominal adipose tissue (AB)	ADIPOQ	0.067*	−0.055	0.164	−0.016	−0.143
		0.735**	0.782	0.403	0.936	0.467
	CYP19A1	−0.132	0.444	0.030	−0.118	−0.073
		0.503	0.018	0.881	0.549	0.710
	VDR	0.005	0.144	0.307	0.140	0.176
		0.981	0.466	0.112	0.479	0.369
	PPARg	−0.011	−0.029	0.249	0.064	−0.278
		0.955	0.885	0.202	0.746	0.152
	MCP	−0.126	0.032	0.197	−0.158	−0.018
		0.523	0.873	0.315	0.423	0.928
Placebo arm (N = 33)						
12-Month gene expression changes in abdominal adipose tissue (AB)	ADIPOQ	0.105	0.144	−0.061	0.004	0.026
		0.562	0.423	0.737	0.982	0.886
	CYP19A1	−0.168	−0.010	0.010	−0.134	−0.064
		0.350	0.956	0.956	0.457	0.724
	VDR	0.013	0.105	−0.117	0.017	0.007
		0.941	0.562	0.517	0.924	0.971
	PPARg	−0.144	0.048	−0.035	−0.073	−0.178
		0.423	0.790	0.848	0.688	0.321
	MCP	−0.155	−0.220	0.241	−0.211	−0.200
		0.388	0.218	0.177	0.239	0.264

* Correlation coefficient; ** P value

*AB* adipose biopsy, *RPFNA* random periareolar fine needle aspiration

In a subgroup analysis limited only to participants receiving vitamin D, women who became replete (i.e., 25(OH)D ≥ 32 ng/mL; *N* = 17) by 12 months showed a small decrease in *MCP-1* expression in breast tissue compared to an increase among women who remained 25(OH)D “insufficient” despite vitamin D supplementation (*N* = 12) (replete: −1.6% vs. non-replete: 61.2%, *p* = 0.015); no statistically significant differences in adipose tissue gene expression were detected (Table 4).

**Table 4:** Twelve-month change in gene expression (unsupervised) according to vitamin D repletion status (25(OH)D</≥32 ng/mL) among women randomized to 2000 IU vitamin D

Gene	All	<32 ng/ml at 12 mo	≥32 ng/ml at 12 mo	p*
AB	*N* = 28	*N* = 11	*N* = 17	
*VDR*	−9.8%	−16.3%	−5.9%	0.611
*CYP19A1*	−17.0%	−7.6%	−24.1%	0.956
*PPARg*	11.6%	10.0%	12.8%	0.239
*Adipoq*	5.7%	15.1%	−0.6%	0.068
*MCP-1*	16.1%	12.3%	19.2%	0.084
RPFNA	*N* = 29	*N* = 12	*N* = 17	
*VDR*	−9.8%	−6.8%	−11.2%	0.435
*CYP19A1*	33.0%	104.1%	−5.3%	0.422
*PPARg*	35.1%	45.8%	29.0%	0.351
*Adipoq*	16.4%	15.4%	17.0%	0.367
*MCP-1*	21.0%	61.2%	−1.6%	0.015

*AB* adipose biopsy, *RPFNA* random periareolar fine needle aspiration

* Based on binary 25(OH)D serum level, adjusted for age, race, baseline serum 25(OH)D, total vitamin D intake (diet + supplement) and sun exposure (h/d)

## Discussion

Weight loss through caloric restriction and exercise has been shown to significantly reduce serum biomarkers of postmenopausal breast cancer risk.^31–33^ However, to our knowledge, this is the first behavioral weight loss intervention study to measure the effect of vitamin D supplementation on gene expression within candidate pathways linking adiposity, vitamin D status and breast cancer risk, as well as the first to study the differential effects of vitamin D administration on gene expression in adipose and breast tissue.

Using samples from both breast RPFNA and subcutaneous adipose tissue we observed no significant difference in gene expression changes during weight loss between women receiving 2000 IU vitamin D compared to placebo, nor were any significant differences in gene expression between groups detected across clinically meaningful categories of weight loss. Furthermore, the 12-month changes in gene expression observed in abdominal adipose tissue compared to breast RPFNA were not strongly correlated. In a post-hoc analysis limited only to women receiving vitamin D, women who became replete (i.e., 25(OH)D ≥ 32 ng/mL) showed a small but significant decrease in *MCP-1* expression in breast RPFNA tissue compared to an increase in women who remained 25(OH)D “insufficient” despite vitamin D supplementation. The observed rise in women who remained vitamin D insufficient was contrary to our hypothesized direction of change but may reflect ongoing local tissue inflammation in our predominantly obese sample, or simply the variability of the gene expression in a small subset of women. Whether the magnitude of attenuation resulting from vitamin D repletion is sufficient to yield meaningful biological effects will require further investigation. These results should be considered hypothesis-generating for future studies but suggest that vitamin D dependent signaling may be tissue specific and dependent on the local availability of coactivators that form complexes with the 1α,25-(OH)_2_ D_3_-VDR/retinoid X receptor (RXR) heterodimer, which then either activates or represses target gene expression.^34^

Our study is among only a few to have tested the effect of vitamin D supplementation on gene expression in the context of human obesity. Mirzaei et al.^35^ measured *VDR* and *PPARG* expression during an 8-week randomized clinical trial in which 94 obese subjects were given daily placebo or 1 mcg alphacalcidol—a vitamin D analog that is rapidly converted to 1,25-dihydroxyvitamin D in the liver. After 8 weeks, the relative expression of *VDR* increased in the alphacalcidol group compared to a relative decrease in the placebo group; *PPARG* gene expression rose 6-fold compared to placebo. In contrast, Wamberg *et al*.^15^ observed reduced mRNA levels of *MCP-1* by 45% (*p* = 0.01), of *IL-6a* and of *IL-8* by 34% (*p* = 0.002 and *p* = 0.03, respectively) in adipose tissue cultures incubated with 1, 25(OH)2D in vitro; yet, observed no similar reductions in adipose tissue inflammation in a concomitant in vivo study using samples of subcutaneous adipose tissue from obese subjects with plasma vitamin D < 20 ng/mL before and after treatment with 7000 IU vitamin D daily (*n* = 22) vs. placebo (*n* = 18) for 26 weeks.

It remains unknown whether vitamin D-related outcomes are more strongly related to achieving a specific magnitude of change in circulating 25(OH)D (e.g., +10 ng/mL) or to a change in status defined by reaching a specific threshold level (e.g., >32 ng/mL). This is an important area for future investigation, as is the potential effect of individualizing vitamin D therapy to repletion at specific levels. There remains no consensus definition for vitamin deficiency and insufficiency, particularly as they related to cancer outcomes.^36^ A dose of 2000 IU may not have been sufficient to significantly alter our outcomes of interest. We may have observed stronger effects using a more conservative definition of insufficiency in our study sample or by including women with serum 25(OH)D concentrations < 10 ng/mL, among whom the effect of vitamin D supplementation could be more pronounced. Other limitations include the fact that we tested only one dose of supplementation and did not test the independent effects of vitamin D without a weight loss intervention. Additionally, the biopsy methods used in the present study yield a mix of cell types. In particular, the RPFNA procedure yields a mix of epithelial and stromal cells, as well as adipose cells and infiltrating macrophages; therefore, in the subset of participants who became vitamin D replete, we cannot discern what cell types were primarily responsible for the change in *MCP-1* without microdissection or analyzing genes specifically expressed by particular cell subtypes. Because our study population was predominantly non-Hispanic white, our results may not be generalizable to women of other racial/ethnic groups. Finally, since biopsies were completed in a subsample of all trial participants and the gene analysis was considered exploratory in our original protocol, we may not have had sufficient power to detect statistically significant effects.

Strengths of our study include its double-blind randomized controlled design, its relatively long duration compared to other vitamin D supplementation studies, the ability to examine the effects of vitamin D supplementation directly in target tissues, and the ability to correlate these changes in gene expression with changes in serum biomarkers.

RPFNA is a minimally invasive, highly reproducible procedure^37^ that has been used to assess short-term risk of breast cancer^38^ and to track cytologic changes in breast epithelial cells, primarily cytologic atypia,^39^ in response to risk reduction strategies.^40, 41^ However, sample yield has been identified as problematic in several cytologic studies,^40–43^ including ours, highlighting the need for the discovery of additional markers, aside from cellular atypia, that can be used to assess changes in breast tissue in response to potential breast cancer prevention interventions. In the 79 samples obtained in our study, only one had insufficient cells for gene expression analysis. Others have also successfully measured gene expression in RPFNA samples.^43–45^ This study supports using gene expression in RPFNA to measure the effects of preventive interventions in breast tissue.

Further study is needed to answer important questions that remain, including those about dose-response and the optimal vitamin D levels required to yield heath-protective benefits, the tissue-specificity of vitamin D’s effects, as well as the timing of vitamin D exposure in relation to cancer etiology. Although this study suggests that vitamin D dependent signaling is tissue specific and that *MCP-1* expression in breast tissue may be favorably altered by achieving higher serum 25(OH)D levels with supplementation, the overall effect of these changes is uncertain. Future studies should incorporate novel markers that can be feasibly and reproducibly sampled in breast tissue in order to more completely elucidate pathways of breast cancer protective effects.

## Methods

The Vitamin D, Diet and Activity (ViDA) study (www.clinicaltrials.gov Identifier NCT01240213), was a 12-month double-blinded, placebo-controlled randomized clinical trial comparing the effects of 2000 IU/day oral vitamin D_3_ (cholecalciferol) supplementation vs. placebo on weight and other biomarkers of breast cancer risk in healthy overweight and obese postmenopausal women with serum 25(OH)D concentrations ≥ 10 and <32 ng/mL (“insufficient”) who were participating in a lifestyle-based weight-loss program.^46^ The accrual goal was 228 women; 218 were randomized. All study procedures were reviewed and approved by the Fred Hutchinson Cancer Research Center Institutional Review Board. All participants provided written Informed Consent.

### Study participants

The main study is described elsewhere.^46^ Briefly, postmenopausal (50–75 years) women with a body mass index (BMI) ≥ 25 kg/m^2^ and serum 25(OH)D concentrations in the insufficient range (10–32 ng/mL) were randomly assigned to 12 months of either: i) 2000 IU/day vitamin D_3_ supplementation + a lifestyle-based weight-loss program (*n* = 109; “Vitamin D”), or ii) daily placebo + a lifestyle-based weight-loss program (*n* = 109; “Placebo”). The primary trial outcome was weight loss; secondary outcomes were changes in body composition (waist circumference, trunk fat, and percentage body fat) and serum biomarkers (insulin and c-reactive protein). Exclusion criteria included: taking >400 IU supplemental vitamin D; renal disease; history of kidney stones; diagnosed diabetes, osteoporosis, or severe congestive heart failure; history of breast cancer or other invasive cancer excluding non-melanoma skin cancer; use of hormone replacement therapy within the past 6 months; alcohol intake >2 drinks/day; current smoking; contraindication to taking 2000 IU vitamin D_3_/day; history of bariatric surgery; current use of weight loss medications; and additional factors that might interfere with the measurement of outcomes or intervention success (e.g., inability to attend facility-based sessions). All participants were offered participation in the tissue marker substudy that included collection of breast tissue through RPFNA and an abdominal subcutaneous fat pad biopsy.

### Randomization

**Fig. 2:**
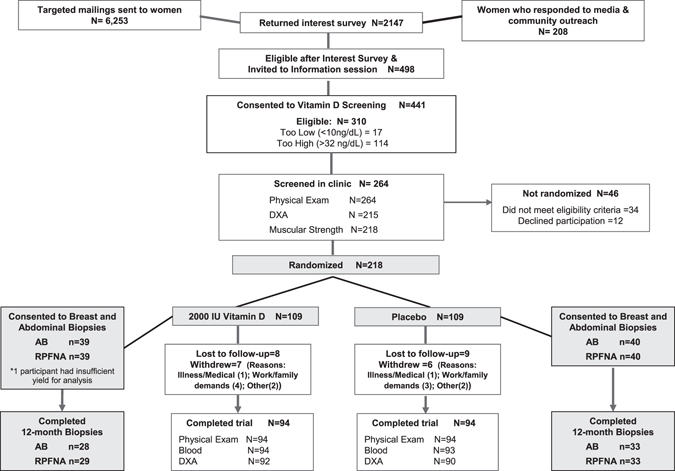
Flow of participants through the Vitamin D, Diet, and Activity (ViDA) study and tissue marker substudy

ViDA participants were randomized by permuted blocks randomization (1:1), stratified according to BMI (<30 or ≥30 kg/m^2^) and consent for the optional breast RPFNA and abdominal fat biopsies. The sample for this study includes the subset of randomized women (*n* = 79) who consented for breast RPFNA and/or abdominal fat biopsies (Fig. 2). All staff except statisticians were blinded to randomization status.

### Interventions

The vitamin D_3_ preparation (2000 IU cholecalciferol) and matching placebo (sunflower oil) gel capsules were created and bottled by J.R. Carlson Laboratories, Inc. (Arlington, IL) as previously described.^46^ In the subset of participants with complete pill counts (Vitamin D: 54%, Placebo: 56%), pill compliance was 98 and 96% among women randomized to vitamin D and placebo, respectively.

The ViDA weight loss program included both a diet and exercise component adapted from a successful intervention that we have used previously, based on the Diabetes Prevention Program and Look Ahead lifestyle change weight loss programs.^47^ The goals of the diet program were: total daily energy intake of 1200–2000 kcal/day based on baseline weight, less than 30% daily energy intake from fat, and a 10% reduction in body weight. The goal of the exercise program was: >45 min of moderate-to-vigorous intensity exercise, 5 days per week (225 min/week) for 12 months. Sessions were performed in our supervised exercise facility and at home.^46^

### Study measures and data collection

Demographic information, medical history, health habits, reproductive and body weight history, dietary intake and supplement use, physical activity, habitual sun exposure, anthropometric measures and body composition were collected at baseline (pre-randomization) and at 12 months.^46^ Circulating 25 (OH)D, serum insulin, c-reactive protein (CRP), adiponectin, leptin and inflammatory cytokines (TNF-α, IL-6, IL-8, IL-10) were measured as previously described.^14, 46^ Estrone and estradiol were quantified by specific radioimmunoassays after organic solvent extraction and Celite column partition chromatography^48, 49^ at the Reproductive Endocrine Research Laboratory (University of Southern California, CA).

### Tissue biopsies and RNA preparation

RPFNA was performed by a trained physician or physician’s assistant according to a method that has been described elsewhere.^38^ In brief, lidocane was used to anesthetize the skin and deeper subcutaneous tissue at two areas in the breast (10:00 and 2:00 positions). Approximately 8 to 10 aspirations were performed using a 1.5-inch 21-gauge needle attached to a 10–12-mL syringe, prewetted with tissue culture medium; half done through the upper outer quadrant site and half through the upper inner quadrant site. Aspirate fluid from all sites was pooled, gently agitated, and placed in RNAlater TissueProtect Tubes (Qiagen Inc) containing 1.5 ml of RNAlater stabilization reagent before being flash-frozen and the stored at −80 °C. Samples (*N* = 144) were subsequently thawed briefly (1 min) in a 37 °C water bath before total RNA extraction.

Immediately following the RPFNA procedure, abdominal subcutaneous adipose-tissue samples (300–500 mg) (*N* = 141) were collected by needle aspiration biopsy approximately 2 inches lateral of the umbilicus under local anesthesia.^50^ Tissue was flash-frozen and stored at −80 °C.

Total RNA was subsequently extracted from RPFNA samples using Qiagen RNeasy Micro kit (Qiagen, Valencia, CA) and from abdominal adipose tissue samples using the Qiagen RNeasy lipid tissue mini kit (Qiagen, Valencia, CA). RNA was quantified using Ribogreen (Invitrogen, Carlsbad, CA) and RNA integrity was assessed on a Bioanalyzer 2100 (Agilent, Santa Clara, CA).

#### Quantitative real-time polymerase chain reaction (qRT-PCR)

Five genes were selected for qRT-PCR: *CYP19A1*, *PPARG*, *ADIPOQ*, *MCP-1*, and *VDR*. In addition, three housekeeping genes *18srRNA*, beta-2-microgobulin (*B2B*), and Beta Glucuronidase (*GUSB*) were measured for normalization across samples.

Fifty ng of each RPFNA RNA and 100 ng of each adipose tissue biopsy RNA was reverse transcribed using the Life Technologies High Capacity cDNA reverse transcription kit (P/N 4374966). Following reverse transcription, the cDNA was used in a preamplification reaction (using Taqman PreAmp Master Mix) and the mRNA expression levels were determined using 2x Gene Expression Master Mix (Applied Biosystems) and pre-developed, inventoried Taqman gene expression assays, specifically assay Hs00240671_m1 (*CYP19A1*); Hs01115513_m1 (*PPARG*); Hs00605917_m1 (*ADIPOQ*); Hs00234140_m1 (*MCP-1*) and Hs00172113_m1 (*VDR*). Samples were batched together and reactions were carried out in triplicate on an ABI 7900HT real-time PCR system. Relative expression levels were calculated using the ∆∆Ct method. Sufficient RNA was available from all samples to measure expression levels of all five genes of interest.

Assuming 80% power and intra-individual correlation of 0.85 in this pilot study sample, we calculated minimal detectable absolute differences in gene expression between study arms of 29 and 30% for VDR, 27 and 41% for CYP19A1, 10 and 21% for PPARG, 9 and 21% for ADIPOQ, and 17 and 50% for MCP-1, in abdominal adipose and breast RPBFA tissue, respectively.

### Statistical analyses

The mean 12-month changes in gene expression in the vitamin D group were compared to placebo using the generalized estimating equations (GEE) modification of linear regression to account for intra-individual correlation over time. Models were adjusted for age, race/ethnicity (white, other), baseline serum 25(OH)D, total vitamin D intake (diet + non-study supplement), and average sun exposure (h/day). The GEE approach for mixed-model regression using the available data was applied to address missing data.

Changes in gene expression were also compared according to whether or not women receiving vitamin D achieved our pre-study definition of repletion (25(OH)D > 32 ng/mL). Pearson correlation coefficients were calculated between 12-month gene expression changes in breast RPFNA tissue compared to abdominal adipose tissue.

Supplemental analyses calculated Pearson correlation coefficients between gene expression and blood analytes (estradiol, estrone, insulin, CRP, adiponectin, leptin, TNF-α, IL-6, IL-8, 1L-10), both for baseline and change from baseline to 12 months, and examined changes in gene expression by degree of weight loss categorized according to clinically meaningful categories: no change/gained weight (referent); lost < 5% of baseline weight; lost > 5–<10% of baseline weight; or lost > 10% of baseline weight.^47^

All statistical tests were two-sided; statistical analyses were performed using SAS software (version 9.4, SAS Institute Inc., Cary, NC).

## Electronic supplementary material

Supplemental Table 1

Supplemental Table 2

## References

[1] Renehan AG, Tyson M, Egger M, Heller RF, Zwahlen M (2008). Body-mass index and incidence of cancer: a systematic review and meta-analysis of prospective observational studies. Lancet..

[2] van Kruijsdijk RC, van der Wall E, Visseren FL (2009). Obesity and cancer: the role of dysfunctional adipose tissue. Cancer. Epidemiol. Biomarkers. Prev..

[3] Renehan AG, Roberts DL, Dive C (2008). Obesity and cancer: pathophysiological and biological mechanisms. Arch. Physiol. Biochem..

[4] Brock K (2010). Low vitamin D status is associated with physical inactivity, obesity and low vitamin D intake in a large US sample of healthy middle-aged men and women. J. Steroid. Biochem. Mol. Biol..

[5] Martins D (2007). Prevalence of cardiovascular risk factors and the serum levels of 25-hydroxyvitamin D in the United States: data from the Third National Health and Nutrition Examination Survey. Arch. Intern. Med..

[6] Kim Y, Je Y (2014). Vitamin D intake, blood 25(OH)D levels, and breast cancer risk or mortality: a meta-analysis. Br. J. Cancer..

[7] Bauer SR, Hankinson SE, Bertone-Johnson ER, Ding EL (2013). Plasma vitamin D levels, menopause, and risk of breast cancer: dose-response meta-analysis of prospective studies. Medicine..

[8] Wang D, Velez de-la-Paz OI, Zhai JX, Liu DW (2013). Serum 25-hydroxyvitamin D and breast cancer risk: a meta-analysis of prospective studies. Tumour Biol..

[9] Gandini S., et al. Meta-analysis of observational studies of serum 25-hydroxyvitamin D levels and colorectal, breast and prostate cancer and colorectal adenoma. *Int J Cancer*. **128**(6), 1414–1424 (2011). 10.1002/ijc.25439 20473927

[10] Ghavamzadeh S, Mobasseri M, Mahdavi R (2014). The effect of Vitamin D supplementation on adiposity, blood glycated hemoglobin, serum leptin and tumor necrosis factor-alpha in type 2 diabetic patients. Int. J. Prev. Med..

[11] Breslavsky A (2013). Effect of high doses of vitamin D on arterial properties, adiponectin, leptin and glucose homeostasis in type 2 diabetic patients. Clin. Nutr..

[12] Nagpal J, Pande JN, Bhartia A (2009). A double-blind, randomized, placebo-controlled trial of the short-term effect of vitamin D3 supplementation on insulin sensitivity in apparently healthy, middle-aged, centrally obese men. Diabet. Med..

[13] Sung CC, Liao MT, Lu KC, Wu CC (2012). Role of vitamin D in insulin resistance. J. Biomed. Biotechnol..

[14] Duggan C (2015). Effect of vitamin D3 supplementation in combination with weight Loss on inflammatory biomarkers in postmenopausal women: a randomized controlled trial. Cancer Prev. Res..

[15] Wamberg L., et al. Effects of vitamin D supplementation on body fat accumulation, inflammation, and metabolic risk factors in obese adults with low vitamin D levels - results from a randomized trial. *Eur J Intern Med.* **24**(7), 644–649 (2013). 10.1016/j.ejim.2013.03.005 23566943

[16] Lopes N (2010). Alterations in vitamin D signalling and metabolic pathways in breast cancer progression: a study of VDR, CYP27B1 and CYP24A1 expression in benign and malignant breast lesions. BMC. Cancer..

[17] Welsh J (2007). Targets of vitamin D receptor signaling in the mammary gland. J. Bone. Miner. Res..

[18] Campbell FC, Xu H, El-Tanani M, Crowe P, Bingham V (2010). The yin and yang of vitamin D receptor (VDR) signaling in neoplastic progression: operational networks and tissue-specific growth control. Biochem. Pharmacol..

[19] Ingraham BA, Bragdon B, Nohe A (2008). Molecular basis of the potential of vitamin D to prevent cancer. Curr. Med. Res. Opin..

[20] Welsh J (2007). Vitamin D and prevention of breast cancer. Acta. Pharmacol. Sin..

[21] Krishnan AV, Swami S, Feldman D (2012). The potential therapeutic benefits of vitamin D in the treatment of estrogen receptor positive breast cancer. Steroids..

[22] Colston KW, Berger U, Coombes RC (1989). Possible role for vitamin D in controlling breast cancer cell proliferation. Lancet..

[23] Zemel MB, Sun X (2008). Calcitriol and energy metabolism. Nutr. Rev..

[24] Mutt SJ, Hypponen E, Saarnio J, Jarvelin MR, Herzig KH (2014). Vitamin D and adipose tissue-more than storage. Front. Physiol..

[25] Gandini S, Gnagnarella P, Serrano D, Pasquali E, Raimondi S (2014). Vitamin D receptor polymorphisms and cancer. Adv. Exp. Med. Biol..

[26] Key T (2002). Endogenous sex hormones and breast cancer in postmenopausal women: reanalysis of nine prospective studies. J. Natl. Cancer. Inst..

[27] Carter JC, Church FC (2009). Obesity and breast cancer: the roles of peroxisome proliferator-activated receptor-gamma and plasminogen activator inhibitor-1. PPAR. Res..

[28] Apostoli AJ (2015). Opposing roles for mammary epithelial-specific PPARgamma signaling and activation during breast tumour progression. Mol. Cancer..

[29] Arendt LM (2013). Obesity promotes breast cancer by CCL2-mediated macrophage recruitment and angiogenesis. Cancer. Res..

[30] Korner A (2007). Total and high-molecular-weight adiponectin in breast cancer: in vitro and in vivo studies. J. Clin. Endocrinol. Metab..

[31] Campbell KL (2012). Reduced-calorie dietary weight loss, exercise, and sex hormones in postmenopausal women: randomized controlled trial. J. Clin. Oncol..

[32] Mason C (2011). Dietary weight loss and exercise effects on insulin resistance in postmenopausal women. Am. J. Prev. Med..

[33] Imayama I (2012). Effects of a caloric restriction weight loss diet and exercise on inflammatory biomarkers in overweight/obese postmenopausal women: a randomized controlled trial. Cancer. Res..

[34] Haussler MR (2013). Molecular mechanisms of vitamin D action. Calcif. Tissue. Int..

[35] Mirzaei K (2014). Insulin resistance via modification of PGC1alpha function identifying a possible preventive role of vitamin D analogues in chronic inflammatory state of obesity. A double blind clinical trial study. Minerva. Med..

[36] Lazzeroni M, Serrano D, Pilz S, Gandini S (2013). Vitamin D supplementation and cancer: review of randomized controlled trials. Anticancer. Agents. Med. Chem..

[37] Ibarra-Drendall C (2009). Reproducibility of random periareolar fine needle aspiration in a multi-institutional Cancer and Leukemia Group B (CALGB) cross-sectional study. Cancer. Epidemiol. Biomarkers. Prev..

[38] Fabian C (2000). Benign breast tissue sampling for prevention studies. Breast. J..

[39] Zalles CM (2006). Comparison of cytomorphology in specimens obtained by random periareolar fine needle aspiration and ductal lavage from women at high risk for development of breast cancer. Breast. Cancer. Res. Treat..

[40] Hidaka BH (2015). Omega-3 and omega-6 Fatty acids in blood and breast tissue of high-risk women and association with atypical cytomorphology. Cancer Prev. Res..

[41] Vinayak S (2013). A clinical trial of lovastatin for modification of biomarkers associated with breast cancer risk. Breast. Cancer. Res. Treat..

[42] Fabian CJ (2007). Reduction in proliferation with six months of letrozole in women on hormone replacement therapy. Breast. Cancer. Res. Treat..

[43] Euhus D (2011). Tamoxifen downregulates ets oncogene family members ETV4 and ETV5 in benign breast tissue: implications for durable risk reduction. Cancer Prev. Res..

[44] Phillips TA, Fabian CJ, Kimler BF, Petroff BK (2013). Assessment of RNA in human breast tissue sampled by random periareolar fine needle aspiration and ductal lavage and processed as fixed or frozen specimens. Reprod. Biol..

[45] Petroff BK, Phillips TA, Kimler BF, Fabian CJ (2006). Detection of biomarker gene expression by real-time polymerase chain reaction using amplified ribonucleic acids from formalin-fixed random periareolar fine needle aspirates of human breast tissue. Anal. Quant. Cytol. Histol..

[46] Mason C (2014). Vitamin D3 supplementation during weight loss: a double-blind randomized controlled trial. Am. J. Clin. Nutr..

[47] Foster-Schubert KE (2011). Effect of diet and exercise, alone or combined, on weight and body composition in overweight-to-obese postmenopausal women. Obesity.

[48] Goebelsmann U (1979). Serum norethindrone (NET) concentrations following intramuscular NET enanthate injection. Effect upon serum LH, FSH, estradiol and progesterone. Contraception..

[49] Probst-Hensch NM (1999). Aromatase and breast cancer susceptibility. Endocr. Relat. Cancer..

[50] Campbell KL (2009). A pilot study of sampling subcutaneous adipose tissue to examine biomarkers of cancer risk. Cancer Prev. Res..

