# Supplementary material for: Gene expression in breast and adipose tissue after 12 months of weight loss and vitamin D supplementation in postmenopausal women
Source: NPJ Breast Cancer. 2017 Apr 21;3:15. doi: 10.1038/s41523-017-0019-5 (PMC5460115; doi:10.1038/s41523-017-0019-5)
Supplement: Supplementary file 1 — Supplemental Table 1 [file 41523_2017_19_MOESM1_ESM.docx]

**Supplemental Table 1**. Associations (Pearson correlation coefficients) between serum biomarker levels and gene expression at baseline.

|  | **Gene expression at baseline (n=78)** | | | | | | | | | | | | | |
| --- | --- | --- | --- | --- | --- | --- | --- | --- | --- | --- | --- | --- | --- | --- |
| **Serum** | **ADIPOQ** | |  | **CYP19A1** | |  | **VDR** | |  | **PPARg** | |  | **MCP-1** | |
|  | AB | RPFNA |  | AB | RPFNA |  | AB | RPFNA |  | AB | RPFNA |  | AB | RPFNA |
| Leptin | **-0.391*** | -0.089 |  | **0.328** | **0.370** |  | 0.085 | 0.133 |  | **-0.275** | -0.071 |  | 0.223 | 0.050 |
| (ng/mL) | **<.001**** | 0.437 |  | **0.003** | **0.001** |  | 0.461 | 0.244 |  | **0.015** | 0.535 |  | 0.050 | 0.662 |
| Adiponectin | **0.252** | 0.149 |  | **-0.305** | **-0.240** |  | -0.194 | -0.061 |  | **0.25** | 0.095 |  | **-0.364** | -0.031 |
| (μg/mL) | **0.026** | 0.193 |  | **0.007** | **0.035** |  | 0.089 | 0.597 |  | **0.027** | 0.408 |  | **0.001** | 0.788 |
| IL-6 | 0.028 | -0.134 |  | 0.00 | -0.059 |  | 0.076 | 0.080 |  | 0.105 | -0.119 |  | 0.016 | -0.052 |
| (pg/ml) | 0.809 | 0.241 |  | 0.997 | 0.608 |  | 0.508 | 0.485 |  | 0.359 | 0.301 |  | 0.891 | 0.653 |
| TNF-a | **-0.226** | 0.036 |  | 0.096 | 0.114 |  | 0.003 | -0.027 |  | **-0.245** | -0.007 |  | -0.059 | 0.053 |
| (pg/ml) | **0.047** | 0.752 |  | 0.402 | 0.322 |  | 0.978 | 0.818 |  | **0.031** | 0.949 |  | 0.606 | 0.647 |
| IL-10 | -0.033 | -0.147 |  | -0.114 | 0.036 |  | -0.077 | 0.007 |  | 0.038 | -0.126 |  | 0.016 | -0.117 |
| (pg/ml) | 0.775 | 0.198 |  | 0.321 | 0.753 |  | 0.501 | 0.952 |  | 0.739 | 0.273 |  | 0.888 | 0.307 |
| IL-8 | -0.001 | 0.024 |  | 0.213 | 0.040 |  | 0.070 | 0.041 |  | 0.018 | -0.001 |  | 0.106 | -0.026 |
| (pg/ml) | 0.994 | 0.834 |  | 0.061 | 0.726 |  | 0.545 | 0.720 |  | 0.874 | 0.995 |  | 0.354 | 0.821 |
| Estradiol | -0.216 | **-0.334** |  | **0.272** | **0.269** |  | 0.025 | 0.095 |  | -0.107 | **-0.345** |  | 0.174 | -0.133 |
| (pg/ml) | 0.057 | **0.003** |  | **0.016** | **0.017** |  | 0.825 | 0.406 |  | 0.350 | **0.002** |  | 0.128 | 0.245 |
| Estrone | -0.170 | **-0.299** |  | 0.19 | **0.244** |  | 0.04 | 0.065 |  | -0.118 | **-0.328** |  | 0.118 | -0.162 |
| (pg/ml) | 0.138 | **0.008** |  | 0.096 | **0.031** |  | 0.726 | 0.570 |  | 0.303 | **0.003** |  | 0.302 | 0.157 |
| Insulin | **-0.296** | -0.202 |  | **0.313** | **0.323** |  | 0.14 | 0.089 |  | -0.137 | -0.152 |  | **0.470** | 0.022 |
| (µu/ml) | **0.009** | 0.076 |  | **0.005** | **0.004** |  | 0.223 | 0.437 |  | 0.233 | 0.183 |  | **<.001** | 0.846 |
| CRP | **-0.357** | -0.185 |  | 0.203 | **0.248** |  | 0.031 | -0.001 |  | **-0.390** | -0.191 |  | **0.269** | 0.093 |
| (mg/ml) | **0.001** | 0.105 |  | 0.075 | **0.028** |  | 0.787 | 0.994 |  | **0.00** | 0.095 |  | **0.017** | 0.421 |
|  | **12-mo gene expression changes (n=62)** | | | | | | | | | | | | | |
|  | **ADIPOQ** | |  | **CYP19A1** | |  | **VDR** | |  | **PPARg** | |  | **MCP-1** | |
|  | AB | RPFNA |  | AB | RPFNA |  | AB | RPFNA |  | AB | RPFNA |  | AB | RPFNA |
| Leptin | -0.097* | -0.059 |  | -0.042 | 0.126 |  | -0.048 | -0.090 |  | -0.150 | -0.064 |  | -0.169 | 0.090 |
| (ng/mL) | 0.456** | 0.648 |  | 0.747 | 0.328 |  | 0.713 | 0.485 |  | 0.250 | 0.623 |  | 0.194 | 0.488 |
| Adiponectin | 0.081 | -0.049 |  | -0.160 | -0.115 |  | -0.127 | 0.044 |  | 0.159 | 0.017 |  | 0.169 | -0.118 |
| (μg/mL) | 0.534 | 0.708 |  | 0.219 | 0.372 |  | 0.328 | 0.733 |  | 0.221 | 0.893 |  | 0.193 | 0.360 |
| IL-6 | 0.068 | -0.092 |  | **0.270** | -0.067 |  | 0.048 | 0.146 |  | 0.253 | -0.118 |  | -0.053 | -0.163 |
| (pg/ml) | 0.608 | 0.481 |  | **0.037** | 0.609 |  | 0.715 | 0.262 |  | 0.052 | 0.367 |  | 0.689 | 0.209 |
| TNF-a | 0.024 | 0.025 |  | 0.136 | 0.148 |  | -0.055 | -0.020 |  | -0.053 | 0.001 |  | 0.056 | 0.200 |
| (pg/ml) | 0.858 | 0.846 |  | 0.298 | 0.254 |  | 0.678 | 0.881 |  | 0.685 | 0.996 |  | 0.673 | 0.123 |
| IL-10 | 0.013 | -0.087 |  | 0.063 | -0.053 |  | -0.11 | -0.025 |  | 0.067 | -0.101 |  | -0.030 | -0.128 |
| (pg/ml) | 0.921 | 0.506 |  | 0.633 | 0.684 |  | 0.401 | 0.846 |  | 0.611 | 0.439 |  | 0.821 | 0.327 |
| IL-8 | **0.271** | -0.067 |  | 0.114 | -0.047 |  | 0.066 | 0.217 |  | **0.266** | -0.112 |  | -0.020 | -0.187 |
| (pg/ml) | **0.036** | 0.606 |  | 0.388 | 0.718 |  | 0.615 | 0.093 |  | **0.04** | 0.391 |  | 0.879 | 0.150 |
| Estradiol | -0.032 | -0.026 |  | 0.052 | 0.248 |  | -0.17 | 0.013 |  | -0.080 | -0.056 |  | **0.276** | 0.223 |
| (pg/ml) | 0.804 | 0.838 |  | 0.693 | 0.052 |  | 0.189 | 0.923 |  | 0.540 | 0.664 |  | **0.031** | 0.082 |
| Estrone | 0.057 | -0.036 |  | 0.106 | 0.123 |  | **-0.292** | 0.077 |  | -0.007 | -0.066 |  | 0.136 | 0.128 |
| (pg/ml) | 0.664 | 0.780 |  | 0.416 | 0.340 |  | **0.022** | 0.554 |  | 0.955 | 0.610 |  | 0.296 | 0.320 |
| Insulin | -0.155 | 0.060 |  | 0.001 | 0.123 |  | -0.016 | -0.090 |  | -0.169 | 0.075 |  | -0.104 | 0.027 |
| (µu/ml) | 0.234 | 0.645 |  | 0.995 | 0.342 |  | 0.900 | 0.485 |  | 0.192 | 0.562 |  | 0.425 | 0.837 |
| CRP | -0.178 | -0.017 |  | **0.456** | 0.015 |  | **0.527** | 0.028 |  | -0.008 | 0.028 |  | 0.084 | -0.025 |
| (mg/ml) | 0.169 | 0.898 |  | **<.001** | 0.911 |  | **<.001** | 0.830 |  | 0.949 | 0.828 |  | 0.522 | 0.849 |
| *Correlation coefficient | |  |  |  |  |  |  |  |  |  |  |  |  |  |
| ** P value. |  |  |  |  |  |  |  |  |  |  |  |  |  |  |
