# Supplementary material for: Gene expression in breast and adipose tissue after 12 months of weight loss and vitamin D supplementation in postmenopausal women
Source: NPJ Breast Cancer. 2017 Apr 21;3:15. doi: 10.1038/s41523-017-0019-5 (PMC5460115; doi:10.1038/s41523-017-0019-5)
Supplement: Supplementary file 2 — Supplemental Table 2 [file 41523_2017_19_MOESM2_ESM.docx]

Supplemental Table 2. Change (%) in gene expression (unsupervised) by degree of weight loss (all women combined).

|  |  |  |  |  |  |  |
| --- | --- | --- | --- | --- | --- | --- |
| **Gene** | **All participants** | **Weight Change Group** | | | |  |
|  |  | **Gained Weight/No change** | **<5% weight loss** | **5-<10% weight loss** | **>10% Weight loss** | ***p*_trend_^a^** |
|  | **N=78** | **N=14** | **N=20** | **N=19** | **N=19** |  |
| **AB** |  |  |  |  |  |  |
| VDR | -9.48% | 14.43% | -10.08% | -11.16% | -3.17% | 0.978 |
| CYP19A1 | -17.66% | 22.00% | 2.35% | -25.94% | -35.55% | 0.779 |
| PPARg | 8.17% | 4.27% | 1.99% | 2.05% | 19.70% | 0.801 |
| Adipoq | 2.19% | 4.59% | -6.19% | -1.58% | 12.46% | 0.121 |
| MCP-1 | 28.38% | -0.64% | 36.96% | 46.94% | 27.41% | 0.623 |
| **RPFNA** |  |  |  |  |  |  |
| VDR | -9.98% | 16.68% | 1.65% | -35.83% | -2.87% | 0.168 |
| CYP19A1 | 10.16% | -3.05% | 90.24% | 17.59% | -17.81% | 0.834 |
| PPARg | 27.78% | 3.45% | 57.21% | 13.09% | 17.47% | 0.556 |
| Adipoq | 9.29% | -6.64% | 21.88% | -5.31% | 10.88% | 0.545 |
| MCP-1 | 1.09% | 10.45% | 13.51% | 74.14% | -40.65% | 0.335 |
| AB=Adipose Biopsy; RPFNA=Random Periareolar Fine Needle Aspiration | | | | | |  |
| *Percentage change of mean of gene expression from baseline visit to 12 month follow up. | | | | | | |
| ^a^Adjusted for age, race, baseline serum 25(OH)D, total vitamin D intake (diet+supplement) and sun exposure (h/d). | | | | | | |
